# Hepatocyte transplantation and advancements in alternative cell sources for liver-based regenerative medicine

**DOI:** 10.1007/s00109-018-1638-5

**Published:** 2018-04-24

**Authors:** Charlotte A Lee, Siddharth Sinha, Emer Fitzpatrick, Anil Dhawan

**Affiliations:** 1 Dhawan Lab, Institute of Liver Studies, King’s College London at King’s College Hospital NHS Foundation trust, London, UK; 2 Paediatric Liver GI and Nutrition Centre, King’s College London at King’s College Hospital NHS Foundation Trust, London, UK

**Keywords:** Hepatocyte transplantation, Induced pluripotent stem cells, Fibroblast, Mesenchymal stem/stromal cell, Hepatic progenitor cells, Liver regeneration

## Abstract

Human hepatocyte transplantation has been actively perused as an alternative to liver replacement for acute liver failure and liver-based metabolic defects. Current challenges in this field include a limited cell source, reduced cell viability following cryopreservation and poor engraftment of cells into the recipient liver with consequent limited life span. As a result, alternative stem cell sources such as pluripotent stem cells, fibroblasts, hepatic progenitor cells, amniotic epithelial cells and mesenchymal stem/stromal cells (MSCs) can be used to generate induced hepatocyte like cells (HLC) with each technique exhibiting advantages and disadvantages. HLCs may have comparable function to primary human hepatocytes and could offer patient-specific treatment. However, long-term functionality of transplanted HLCs and the potential oncogenic risks of using stem cells have yet to be established. The immunomodulatory effects of MSCs are promising, and multiple clinical trials are investigating their effect in cirrhosis and acute liver failure. Here, we review the current status of hepatocyte transplantation, alternative cell sources to primary human hepatocytes and their potential in liver regeneration. We also describe recent clinical trials using hepatocytes derived from stem cells and their role in improving the phenotype of several liver diseases.

## Introduction

The liver is responsible for a diverse range of functions within the body ranging from xenobiotic metabolism to plasma protein synthesis [1]. However, the liver can become susceptible to acute failure (ALF) due to viral infection or drug effect amongst other aetiologies [2]. Liver-based metabolic disorders that are caused by single-gene defects can result in a lack of specific liver-based enzyme function which may result in damage to other organs most often irreversible neurodisability [3]. Orthotopic liver transplantation (OLT) is currently the only viable treatment for severe ALF and certain liver-based metabolic disorders [2, 4, 5]. Liver replacement is not futuristic for liver-based metabolic defects where gene therapy would be ideal. Notably, a shortage in healthy donor livers has led to research into alternative treatment options [6]. Hepatocyte transplantation (HT), where liver cells may provide a potential alternative to OLT or act as a bridge until a suitable organ becomes available, has been demonstrated, with over 100 cases published worldwide showing the safety and preliminary efficacy of the technique [7, 8].

This review will focus on the current status of HT, alternate cell sources to primary human hepatocytes and alternate methods of liver regeneration which can be used either independently or in combination with HT.

## The current status of hepatocyte transplantation

HT is a less invasive alternative to OLT. Hepatocytes are isolated from donor livers that have been rejected for organ transplantation in view of prolonged warm or cold ischaemia times, mild steatosis or aberrant anatomy. Hepatocytes isolated from one donor liver can yield a high quantity of cells and be used to treat multiple patients [9, 10]. The ability to cryopreserve these cells is one of the major advantages of HT, potentially providing an off-the-shelf treatment and enabling cells of all blood groups to be immediately available for emergency cases, which is particularly important for patients with ALF. Several routes of infusion for cell transplantation have been described including intraportal, intrasplenic and intraperitoneal. Depending on the age of the patient, infusion of hepatocytes into the portal vein can be undertaken via the umbilical vein under fluoroscopy to avoid a patent ductus venous. In older children, radiological or surgical catheters are required using radiological guidance [11–13].

Hepatocyte transplantation can be used to treat patients with liver-based metabolic diseases and ALF [7, 14, 15]. Metabolic liver diseases treated using HT include Crigler-Najjar syndrome type 1 (CN-1), urea cycle defects and factor VII (fVII) deficiency [16]. Crigler-Najjar syndrome has an incidence of 1 in 1,000,000 births in the UK, and although the primary long-term treatment is liver transplantation, the lack of an appropriately sized, blood matched donor organ can lead to irreversible neurological disability. There are reports of 8 patients that have received between 1.4 and 7.5 billion hepatocytes, which showed up to a 50% reduction in bilirubin and UGT activity and a decrease in the need for phototherapy. The majority of these patients went onto receive an OLT within a year as the effects of HT were not long lasting, with one patient avoiding OLT for 4 years [16–21].

Several urea cycle defects have also been shown to benefit from hepatocyte infusions. This includes ornithine transcarbamylase deficiency (OTC), which is an X-linked genetic condition affecting males that causes hyperammonemia and can also lead to neurological implications including developmental delay and learning disabilities. HT led to decreased ammonia levels and increased urea production in six of these patients, with four receiving an OLT within 7 months and two reported mortalities [14, 22–25]. Other metabolic liver diseases shown to improve following HT include familial hypercholesterolemia where there was up to a 20% decrease in LDL in three patients, fVII deficiency where there was a 70% decrease in the need for recombinant fVII for 6 months and ASL deficiency where the was a decrease in ammonia production and increased psychomotor abilities [26–28]. Nevertheless, the long-term benefits of HT for the treatment of patients with liver-based metabolic disease have yet to be shown.

HT can also be used in patients with ALF. Although liver transplantation is the primary treatment, paediatric patients may die whilst waiting for an appropriately size and blood-matched liver, with no other treatment being shown to improve survival [29]. Furthermore, existing listing criteria are not robust in terms of accurately predicting death and survival; hence, some patients receive a liver transplantation, even if their liver may spontaneously recover, leading to unnecessary surgeries with life-long immunosuppression. Thirty-seven patients with acute liver failure have received human hepatocytes for both drug induced, viral and idiopathic ALF. Ten of these patients underwent intraportal hepatocyte infusions, with two making a full recovery without the need for OLT and three were successfully bridged to transplantation [15, 30–33]. However, there is a high risk associated with placing intrahepatic invasive catheters in coagulopathic patients. As an alternative, it is possible to encapsulate hepatocytes in alginate microbeads made from purified alginate using an encapsulator [34]. The semi-permeable membrane within the microbeads allows exchange of metabolites, maintaining synthetic detoxification function, whilst also protecting against immunocompetent cells by preventing the entry of antibodies [35, 36]. Hepatocyte-containing microbeads are transplanted into the intraperitoneal cavity. The transplanted hepatocytes can assist the necessary liver functions, allowing the liver to recover. After full recovery, usually 1 month, the microbeads can be removed using a laparoscopic peritoneal lavage.

**Fig. 1:**
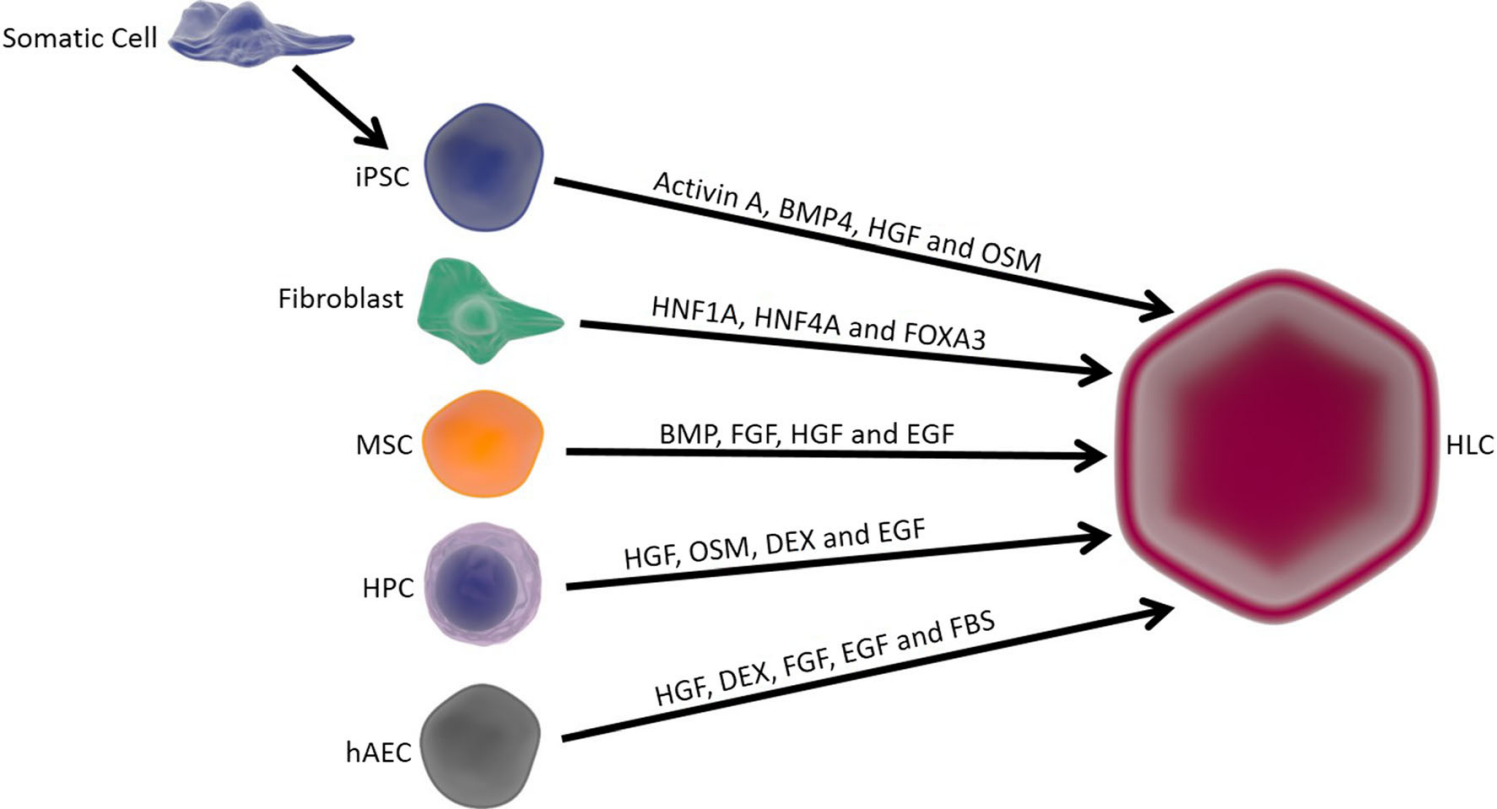
Potential alternative cell sources (induced pluripotent stem cells, fibroblasts, mesenchymal stem/stromal cells and hepatic progenitor cells) which can be used to generate hepatocytes. Gene transfer is used to convert somatic cells to iPSCs and fibroblasts to HLCs. All other transformations occur under culture conditions. *HLC* induced hepatocyte, *iPSC* induced pluripotent stem cells, *MSC* mesenchymal stem cells, *HPC* hepatic progenitor cells, *hAEC* human amniotic epithelial cells, *BMP* bone morphogenetic protein, *OSM* oncostatin M, *HGF* hepatic growth factor, *HNF1A* hepatocyte nuclear factor 1 homeobox alpha, *HNF4A* hepatocyte nuclear factor 4 alpha, *FGF* fibroblast growth factor, *EGF* epidermal growth factor, *Dex* dexamethasone, *FBS* foetal bovine serum

Despite extensive clinical data, long-term clinical outcomes of HT have yet to be established with any type of liver-based metabolic disease or ALF. There are still many limitations to the technique, and current research aims to overcome this. Firstly, the cryopreservation process requires further optimisation with the current protocol resulting in low viability and function of hepatocytes post-thawing. Furthermore, transplanted donor hepatocytes undergo immune rejection with up to 70% of engrafted cells cleared within the first 24 h post-transplantation [37]. Upon transplantation, donor hepatocytes are recognised and activate the instant blood-mediated inflammatory reaction, during which both the complement and coagulation pathways are activated. Innate immune cells such as Kupffer cells, natural killer cells and monocytes rapidly clear transplanted donor hepatocytes [38, 39]. A major limitation of HT is the lack of good quality donor organs from which to isolate cells from. Neonatal livers have been investigated as a potential cell source as they are infrequently used for OLT due to the high incidence of hepatic artery thrombosis [40]. However, they may be an ideal cell source for HT, with one study demonstrating their mature function of cytochrome P450 and ureagenesis enzymes, with increased attachment efficiency and viability compared to hepatocytes isolated from adult donor livers [41, 42]. Currently, the number of neonatal donations is not sufficient to maintain an active clinical HT programme [41, 43]. As a result, there is a requirement to investigate alternative cell sources to resolve the shortage of healthy donor organs and avoid recipient immune rejection (Fig. 1 and Table 1).

**Table 1:** Summary of selected clinical trials globally, researching the therapeutic benefits of alternative cell sources in liver disease

Alternative cell sources	Advantages	Disadvantages
Induced pluripotent stem cells (iPSC)	• Patient-specific cell generation • Reduced immune response • Disease modelling • Limitless pool of cells	• Inadequate long-term functionality • Potential tumour formation
Fibroblasts	• Patient-specific cell generation • Reduced immune response • Disease modelling	• Proliferation arrest • Residual epigenetic memory • Resistant to transformation
Mesenchymal stem/stromal cells (MSC)	• Patient-specific cell generation • Immune modulation • Easily accessible from several tissues of the body	• Could potentially lose functionality
Hepatic progenitor cells (HPC)	• Naturally differentiate into new hepatocytes • Can be used to generate a number of new hepatocytes • Considered to be involved in hepatocyte regeneration	• Could play no role in liver regeneration • May be involved in the progression of liver fibrosis • Acquired from donor livers so may cause immune rejection • Immunosuppressive drugs would be required
Amniotic epithelial cells (hAEC)	• Reduced risk of tumour formation • Easily accessible • Reduced ethical implications • Reduced immune reaction	• Gene expression similar to foetal cells rather than adult hepatocytes

## Alternative cell sources for hepatocyte transplantation

### Induced pluripotent stem cells generated from somatic cells

Induced pluripotent stem cells (iPSCs) that can be differentiated into hepatocyte-like cells (HLCs) are being widely explored as an alternative to primary human hepatocytes. These cells offer an excellent alternative source of hepatocytes and are advantageous over primary human hepatocytes because of their unlimited cell source and their ability to avoid the immune system [44]. Methods to produce iPSC-derived HLCs are well established, with most protocols using a three-dimensional matrix such as Matrigel® to establish the generation of hepatocytes. However 2D matrices including collagen have also shown successful iPSC to HLC differentiation [45, 46]. An effective protocol for generating HLCs has been established via the use of specific growth factors such as activin A and bone morphogenetic protein 4 (BMP4), which are crucial for initiating the first step of hepatic maturation. Hepatocyte-growth factor (HGF) and oncostatin stimulate hepatoblast formation and hepatocyte-like differentiation, respectively. Si-Tayeb et al. showed that iPSC-derived HLCs displayed hepatic functionality, including glycogen accumulation, lipoprotein uptake and urea synthesis [47]. iPSCs are advantageous over primary human hepatocytes as generation from somatic cells of an individual will prevent activation of the recipient’s immune response, avoiding the use of immunosuppressive drugs [38, 45, 48, 49]. iPSCs can proliferate indefinitely, forming a limitless pool of HLCs allowing patients to receive multiple infusions if necessary [48, 50]. iPSC-derived HLCs can also be used as a model for several metabolic liver diseases including familial hypercholesterolemia, α_1_-antitrypsin deficiency and glycogen storage disease type 1a. By generating cells with similar phenotypes to those caused by inherited diseases, it is possible to study the mechanisms of dysregulated cellular functions and identify ways to treat or reverse the condition [51]. Currently, the functional ability of HLCs is not comparable to human primary hepatocytes. Yu et al. showed that HLCs co-express alpha-fetoprotein and albumin, suggesting that they are not fully mature. Levels of albumin synthesis, urea production, cytochrome p450 activity and mitochondrial function are also significantly lower than human primary hepatocytes [50]. Furthermore, there are genetic variations in donor cells that effect the differentiation propensity of iPSCs which has been attributed to genetic variation of the donor, cell culture conditions and iPSC generation protocols [52, 53]. Following the development of new hepatic differentiation protocols, Kajiwara et al. compared 28 iPSC lines derived from different somatic cells and showed that it was the origin of the donor cells that determined the variation in hepatic differentiation and not the derivation method [53].With albumin used as a marker to assess functionality, HLCs derived from human dermal fibroblast-iPSCs and peripheral blood cell-iPSCs showed minimal variation in hepatic differentiation from the same donor; however, inter-donor hepatic variation was more prominent. This creates a complication when using iPSCs for therapeutic use as non-identical cell lines cannot guarantee the same quality of HLC production [48, 53].

In addition, the tumorigenic potential of these cells due to the presence of oncogenes such as c-Myc may raise a safety concern regarding the clinical application of these cells. Chen et al. reported no formation of teratomas or tumours in Gunn rats transplanted with HLCs, suggesting a loss of pluripotent characteristics within these cells [54]. Although HLCs represent an ideal cell source for HT with no risk of rejection, work is still ongoing to advance their functional capacity and fully validate the safety and efficacy of using these stem cells.

### Fibroblasts

Human fibroblasts offer another potential source of HLCs for HT. Fibroblasts are connective tissue cells found in all areas of the human body [55]. Huang et al. established the first reprogramming of human fibroblasts to hepatocytes using both foetal and adult connective tissue cells. Using lentiviruses as a vector for expressing transcription factors, hepatocyte nuclear factor 1 homeobox alpha (HNF1A), HNF4A and FOXA3, successful fibroblast to HLC transformation has been achieved, with HNF1A being crucial for the human fibroblast reprogramming [56]. Simeonov et al. also achieved fibroblast transformation using exogenous HNF1A messenger RNA (mRNA) [57]. Within the same year, Du et al. derived HLCs from human fibroblasts, with the generated hepatocytes possessing phase I/II/III drug metabolic activity comparable to primary human hepatocytes [58]. Fibroblast-derived HLC production requires only a single transformation step and they can be patient-specific, reducing the chances of immune rejection and avoiding the use of immunosuppressive drugs [38, 49, 56]. Despite these advantageous, fibroblast-derived HLCs have therapeutic limitations. They have a limited reproductive capability and cannot be used for repeat infusions in a single patient. Furthermore, human fibroblasts are resistant to hepatic transdifferentiation, thereby creating an additional barrier when generating HLCs [56]. Hepatocytes generated from reprogrammed fibroblasts may still retain epigenetic memory from the fibroblast cell of origin. This creates limitations when choosing fibroblasts for hepatic transformation, as cells with significant epigenetic differences to hepatocytes may be further resistant to reprogramming and reduced functionality [59].

### Hepatic differentiation of amniotic epithelial cells

It is also possible to generate hepatocytes from amniotic epithelial cells. These cells have stem cell markers such as OCT-4, Nanog, SOX-2 and Rex-1, and as they do not have telomerase reverse transcriptase, they show a stable phenotype without the risk of tumorigenic potential [60]. Such cells have minimal ethical implications, and there is no shortage of placental tissue from which to isolate the cells. Following culture in Matrigel® or liver-derived ECM, these cells had albumin and CYP3A4, 3A7, 2B6 and 2D6 mRNA levels which increased over time with a peak at day 21 [61]. Following transplantation into the SCID mouse, genes were expressed for human cytochrome p450 genes, metabolic enzymes and hepatocyte-enriched transcription factors and plasma proteins 6 months post-transplantation. It has now been suggested that hepatic differentiation of amniotic epithelial cells (hAECs) represent a promising non-controversial, unlimited source of cells for liver-based metabolic diseases.

### The regenerative capacity of resident liver cell hepatic progenitor cells

Hepatic progenitor cells (HPCs), also known as oval cells, are believed to differentiate into mature hepatocytes or cholangiocytes, upon liver damage and help in tissue restoration. HGF and EGF are critical in inducing the transformation of HPC into hepatocytes. HGF activates the MET receptor, which further upregulates the expressions of AKT and STAT3 driving hepatic transformation. A lack of MET receptors completely attenuates HPC to hepatocyte differentiation even in the presence of EGFR. However, EGFR-null HPCs were still able to sufficiently transform into hepatocytes with MET alone [62].

Zhang et al. established in vitro generation of HLCs from human foetal HPCs, under the influence of oncostatin M (OSM), DEX and HGF. These newly differentiated hepatocytes have functional glycogen storage, albumin secretion and cytochrome p450 activity with Khuu et al., suggesting that in vitro differentiated adult hepatic stem cells are also capable of urea production and ammonium chloride metabolism [63, 64]. Unlike some other HLCs, hepatocytes formed from liver-derived progenitor cells have reached clinical application. Sokal et al. transplanted HPCs in a 3-year-old female patient suffering with OTC deficiency. Previous transplantation of cryopreserved hepatocytes failed to improve the patient’s symptoms. Fourteen weeks post-infusion, biopsies showed 3% presence of donor cells and the patient showed some functional improvement with a reduction in disease-related anorexia. Unfortunately, 6 months post-infusion, the child underwent OLT and later died. These results suggest that HPCs could play a role in treatment of metabolic liver disease; however, longer-scale clinical trials are required to assess their full potential [65].

#### Human bile duct cells

In addition to HPCs, mature hepatocytes can be derived from a number of other resident cell types within the liver. Huch et al. established a protocol differentiating primary human bile duct cells (EpCAM^+^) into genetically stable functional HLCs in both in vitro and in vivo transplantations. Organoids were formed using ductal cells, and using medium consisting of BMP7, EGF and HGF, successful hepatic differentiation was achieved. Newly formed HLCs demonstrated albumin production, CYP3A3/4/5 activity and bile acid secretion. Furthermore, organoids successfully engrafted into Balb/c nude mice with induced liver damage, sustaining albumin and α-1-antitrypsin levels for up to 120 days in two out of five recipient mice. Debate remains over the genetic stability of fibroblasts and iPSCs as cell sources for HLCs. The expandable nature and genetic stability of HLCs derived from human bile ductal cells makes them a desirable alternate cell source [66].

#### Chemically induced liver progenitors

Recently, it has been shown that mature hepatocytes convert to HLCs during chronic liver injury [67]. Katsuda et al. showed that a cocktail of small molecules Y-27632, A-83-01 and CHIR99021 could contribute to the induction and maintenance of bipotent chemically induced liver progenitor cells (CLiPs). These cells could either be differentiated into mature hepatocytes or biliary epithelial cells. Rat CLiPs were capable of repopulating immunodeficient mice with chronic liver injury. Albumin levels were used to assess liver functionality, which showed a consistent increase up until 8 weeks post-transplantation. Immunohistochemistry showed that up to 75–90% of the mouse liver had been replaced by rat hepatocytes, demonstrating a selective proliferative advantage for the healthy donor cells. Mouse ductal structures also showed CLiPs-derived cell replacement, displaying the biopotency of the lineage. If similar protocols could be established using human hepatocytes, this offers an additional source for HLCs for use in HT. Furthermore, the bipotent properties of CLiPs suggest that they could be used to tackle diseases related to the biliary tree as well as the liver [68].

## The role of mesenchymal stem/stromal cells in liver-based regenerative medicine

Mesenchymal stem/stromal cells (MSCs) have been investigated as another cell source for hepatocyte differentiation but with limited and controversial results. More promising is their immunogenic effect, and now, MSCs are being investigated as an immunomodulatory therapy to treat liver a number of different liver diseases.

MSCs can be isolated from various tissues, such as bone marrow, adipose tissue and the umbilical cord. BMP and fibroblast growth factor (FGF) induction lead to the differentiation of MSCs to the hepatic lineage, with dexamethasone (DEX) and IL-16 inducing hepatic maturation. Single-step procedures are also commonly used with HGF and epidermal growth factor (EGF). Transformed cells generated from this procedure usually exhibit functional hepatic properties 2–3 weeks post-culturing but do lose functional capabilities when cultured for prolonged periods [69]. Furthermore, it is still debated whether MSC-derived hepatocytes are able to efficiently re-populate a host liver to provide adequate function and clinical application is still in its infancy [70]. Perhaps of greater potential is the immunomodulatory effect of MSCs. MSCs play a key role in immune modulation due to their lack of MHC-I/MHC-II receptors and are unlikely to trigger a T cell response. Furthermore, MSCs are considered to reduce T cell proliferation and cytotoxicity, as well as improving liver injury and enhancing liver regeneration [71, 72]. This immune evasion capability has resulted in MSCs adopting the title of “immunoprivileged” or “immunotolerant” cells [73]. Thus, MSCs could be used in conjunction with hepatocytes during HT to increase engraftment and reduce the immune response. Hwang et al. showed that intrasplenic transplantation of MSC-derived HLCs restored liver functionality in rat models with thioacetamide-induced liver cirrhosis. Naïve implanted MSCs firstly transdifferentiated into hepatic oval cells and later into HLCs. The presence of newly formed HLCs reduced inflammation, reversed fibrosis and repaired damaged hepatocytes. The exact mechanism by which MSCs induce hepatic recovery is unclear, but the authors suggest that activation of humoural factors could contribute to liver regeneration [74].

### Mechanisms of MSCs in liver regeneration

Recently, it has been suggested that MSCs modulate liver failure by several mechanisms including differentiation of MSCs to replace damaged cells, secretion of soluble factors by MSCs to promote liver repair, MSC-mediated transfer of mitochondria by tunnelling nanotubules and by MSC-mediated transfer of proteins, RNA, hormones and chemicals by extracellular vesicles such as exosomes or microvesicles [75].

MSC conditioned medium (MSC-CM) can play an important role in attenuating liver disease with a wide range of soluble factors thought to be present within MSC-CM [76]. Interleukin-6 secreted by MSCs reduces apoptosis in liver injury [77]. Furthermore, MSC secreted TGF-β and nerve growth factor resulted in apoptosis of hepatic stellate cells, a hallmark of liver fibrosis [78, 79]. Huang et al. showed that mice with fulminant hepatic failure (FHF) and chronic liver failure treated with MSCs or MSC-CM displayed reduced liver pathology. Only MSC treatment of FHF mice showed great reduction in pro-inflammatory T helper-1/17 cells and upregulation of T regulatory cells. This indicates that direct presence of MSCs is required to induce complete immunomodulatory effects [80].

In addition to soluble factors present in MSC-CM, recently, exosomes have been identified as an important component that may promote hepatic regeneration. Tan et al. (2014) showed that CCL4-induced liver injury reduced AST and ALT levels and decreased the number of necrotic cells in mice that were treated with MSC-derived exosomes. Furthermore, proliferation of hepatocytes was greater, which was associated with increased expression of proliferating cell nuclear antigen [81]. The authors suggest that MSC-derived exosomes may have a therapeutic potential in toxic liver injury. It has also been suggested that tunnelling nanotubules can form between cells that act as a transport network, allowing the transport of mitochondria and lysosomal vesicles [82]. Currently, the transfer of mitochondria through tunnelling nanotubules from umbilical cord-derived MSCs to hepatocytes is being investigated as a mechanism for their increased survival and function and may play a role in liver regeneration [83].

MSCs represent an ideal cell source for liver regeneration-based medicine due to their easily accessible source, their immunomodulatory properties and their potential of transdifferentiating into hepatocytes.

## Current clinical trials using cell therapy for liver-based diseases

There are now multiple phase I/II and III clinical trials using different types of stem cells to improve a number of liver diseases including cirrhosis, liver failure and liver-based metabolic disorders. In liver cirrhosis and end-stage liver disease, reports have proposed that MSCs can replace hepatocytes in the injured liver, stimulating liver regeneration (Table 2).

**Table 2:** A summary of the advantages and disadvantages of various cell sources which can be used to generate induced hepatocytes for hepatocyte transplantation

Study name	Cell source	Condition	Intervention	Primary outcome	Study phase	Location	Start and end date	References
Umbilical cord mesenchymal stem cell transplantation combined with plasma exchange for patients with liver failure	Umbilical mesenchymal stem cell (UC-MSC)	Liver failure	Conventional treatment only (antiviral drugs, lowering aminotransferase and jaundice medicine) Conventional treatment plus UC-MSC transplantation (via peripheral vein slowly for 30 min 1 × 10^5^/kg, once a week, four times) or plasma exchange (2000 mL every 3 days, three times) or both	Survival rate and time (time frame 48 weeks)	Phase I and II	Department of Infectious Diseases, The Third Affiliated Hospital of Sun Yat-Sen University Guangzhou, Guangdong China	November 2012–March 2015	[84]
Safety and efficacy of human umbilical cord-derived mesenchymal stem cells for treatment of HBV-related liver cirrhosis	Umbilical mesenchymal stem cell (UC-MSC)	Liver cirrhosis End-stage liver disease	Conventional treatment or UC-MSC transplantation (1 × 10^6^ cells/kg via hepatic artery)	One-year survival rate (time frame 1-year treatment)	Phase I and II	Xijing Hospital of Digestive Disease Xi’an, Shaanxi, China	September 2012–September 2015	[85]
Phase II safety study of two dose regimens of HepaStem in patients with ACLF (HEP101)	Human liver-derived mesenchymal stem cell (HepaStem)	Acute-on-chronic liver failure	Low-dose cohort—two dose regimens of HepaStem will be given, differing in cell quantity per infusion. The low dose regimen will be given to the first cohort (first six patients included in the study). High-dose cohort—given to the second cohort after evaluation of the safety of the first cohort (stepwise approach)	Occurrence of adverse events (AEs) up to day 28 of the active study period (time frame up to 28 days post-first infusion day)	Phase II	Hȏpital Erasme, Brussels, Belgium UZ Antwerpen, Edegem, Belgium KU Leuven, Leuven, Belgium CHU de Liège, Liège, Beligium Cliniques St. Luc, Woluwe-Saint Lambert, Belgium Hȏspital Beaujon, Clichy, France Hȏpital de la Croix Rousse, Lyon, France Hȏpital Paul Brousse, Villejuf, France	December 2016–September 2018	[86]
Bone marrow stem cells as a source of allogenic hepatocyte transplantation in homozygous familial hypercholesterolemia	Bone marrow stem cells	Familial hypercholesterolemia	Bone marrow stem cell transplantation. 6 × 10^8^ to 1 × 10^9^ cells infused through the portal vein over 30 min, done once	Serum cholesterol and LDL level (time frame 6 months)	Phase I	Digestive Disease Research Center, Shariati Hospital, North Kargar Ave., Tehran, Iran, Islamic Republic	June 2007–June 2008	[87]
Study to evaluate the efficacy of HepaStem in urea cycle disorders of paediatric patients (HEP002)	Human liver-derived mesenchymal stem cell (HepaStem)	Urea cycle disorders	HepaStem administered in maximum four infusion days, spread over 8 weeks, with 2/3-week interval between infusions. Target total dose 5 × 10^7^/kg body weight	Efficacy as determined by de novo ureagenesis (C13 tracer method) (time frame 6 months post-first infusion day)	Phase II	Cliniques Universitaires Saint-Luc, Brussels, Belgium, Hȏpital Jeanne de Flandre, CHRU Lille, Lille, France Instytut–Pomnik Centrum Zdrowia Dziecka, Warszawa, Poland Hospital Materno Infatil de Badajoz, Badajoz, Spain Hospital Universitari Vall d’Hebron de Barcelona, Barcelona, Spain Hospital Materno Infantil de Málaga, Málaga, Spain	October 2014–March 2017	[88]
Safety and tolerance of immunomodulating therapy with donor-specific MSC in paediatric liver-donor liver transplantation (MYSTEP1)	Bone marrow-derived MSCs	Paediatric liver transplantation	Two doses of 1 × 10^6^ MSCs/kg body weight	Number of participants with MYSTEP-score grade 3 and grade 2 (toxicity of MSC infusion), number of participants with occurrence of any severe adverse events, graft function after liver transplantation, number of participants with abnormal liver tests)	Phase I	University Children’s Hospital, Tubingen, Germany	July 2013–Januray 2019	[89]
Therapeutic strategy and the role of mesenchymal stromal cells for ABO incompatible liver transplantation	Mesenchymal stem cells	Liver transplantation	Six doses of 1 × 10^6^/kg body weight MSCs are given, intravenously	Efficacy 1-year graft survival rate	Phase I	The Third Affiliated Hospital, Sun Yat-Sen University, Guangzhou, Guangdong, China	February 2014–March 2017	[90]
Human mesenchymal stem cell transfusion is safe and improves liver function in acute-on-chronic liver failure patients	Umbilical cord mesenchymal stem cell (UC-MSC)	Acute-on-chronic liver failure	Conventional treatment and 0.5 × 10^6^/kg body weight UC-MSCs are given, intravenously at baseline, 4 weeks, and 8 weeks Conventional treatment and saline was used for the control group	Liver functionality tested over 48 weeks 72-week survival rate	Phase I and II	Beijing 302 Hospital Beijing, Beijing, China	March 2009–March 2014	[91]
Safety study of HepaStem for the treatment of urea cycle disorders (UCD) and Crigler-Najjar syndrome (CN) (HEP001)	Human liver-derived mesenchymal stem cell (HepaStem)	Urea cycle disorders Crigler Najjar syndrome	HepaStem low dose 12.5 × 10^6^/kg body weight HepaStem intermediate dose 50 × 10^6^/kg body weight HepaStem high dose 200 × 10^6^/kg body weight	Safety of HepaStem in paediatric patients suffering from urea cycle disorder and Crigler-Najjar syndrome	Phase I and II	Saint Luc University Hospital, Brussels, Belgium Universitair Ziekenhuis (US) Antwerpen, Edegem Belgium CHU Bicetre, Le Kremlin Bicȇtre Cedex, France Hȏpital Jeanne de Flandre, CHRU Lille, Lille Cedex, France Hȏpital des Enfants, CHU de Toulouse, Toulouse, Cedex 9, France Rambam Medical Center, Meyer Children’s Hospital, Haifa, Israel Hadassah Ein-Kerem Medical Center of Israel, Jerusalem, Israel Schneider Children’s Medical Center of Israel, Petach Tikva, Israel Ospedale Pediatrico Bambino Gesu di Roma, Roma, Italy Birmingham Children’s Hospital London, London, United Kingdom	March 2012–April 2015	[92]
Macrophage therapy for liver cirrhosis	Autologous macrophages	Liver cirrhosis	Autologous activated macrophages infusion via peripheral vein for 30 min. Standard medical care used as the control	Liver function (MELD score) at 3 months	Phase I and II	Edinburgh Royal Infirmary Little France Crescent Edinburgh EH16 4SA United Kingdom	August 2016–August 2021	[93]

Information from *Clincialtrials.gov* [80]

Shi et al. (2012) showed that transfusion of umbilical cord-MSC (UC-MSC) into 24 patients with acute-on-chronic liver failure showed marked increase in liver functionality when compared to the control of 19 patients transfused with saline. Patients were monitored over 48 weeks, with the treatment group showing an increase in albumin secretion, platelet count and a reduced end-stage liver disease (MELD) score. Furthermore, survival rate after 72 weeks was also higher in the treatment group compared to the control, with 20.8 and 47.4% mortality rate, respectively. The author suggests that although the mechanism of improved liver function may be unclear, in vivo differentiation of UC-MSC into hepatocytes is unlikely due to the short period of hepatic recovery and with only one treatment patient showing increased alpha-fetoprotein levels. It is more likely that soluble factors produced by MSCs may enhance liver revascularization and proliferation [95].

One study has suggested that plasma exchange (PE) helps promote liver regeneration and recovery, leading to UC-MSC differentiation into HLCs. A phase I/II clinical trial is now in progress, transplanting UC-MSCs into patients with liver failure. Patients received either conventional treatment (anti-viral drugs) with UC-MSCs and/or PE treatment, and survival rates were assessed at 48 weeks [84]. For patients with acute-on-chronic liver failure, Promethera Biosciences have developed a product known as HepaStem, which are MSCs that have the potential to differentiate into HLCs. A phase IIa clinical trial is now in progress, transplanting these cells via IV injection to establish the safety and biological efficacy of these cells. Bilirubin, creatinine, INR and albumin values are being assessed at day 28, 2 months and 1 year post-infusion. In addition to using stem cells for liver failure, HLCs are now being used for clinical HT to replace primary hepatocytes in patients with liver-based metabolic disorders. Bone marrow-derived MSCs transdifferentiated into hepatocytes have been transplanted via the portal vein into patients with familial hypercholesterolemia. Serum cholesterol/LDL levels were assessed after 6 months to determine the efficacy of the technique. Furthermore, HepaStem cells are also being used to treat patients suffering from urea cycle disorders. Ureagenesis, ammonia values and amino acid levels are being monitored as well as behaviour, cognitive skills and health-related quality of life indicators for up to 12 months post-infusion [96].

MSCs are also being used clinically for immunomodulating therapy in many liver-based applications. One trial is currently investigating the use of MSCs to promote allograft tolerance and reduce the toxicity that results from exposure to calcineurin inhibitors. Paediatric patients receiving a liver transplantation undergo IV injection of bone marrow-derived MSCs. MSC toxicity is being monitored as well as graft function measured by aminotransferase and gamma glutamyl transferase activity, bilirubin, albumin and INR and the individual need for immunosuppressive medication. In addition, MSCs are being used as immunomodulators in ABO-incompatible liver transplantation. The study aims to determine if MSCs are safe and effective at reducing the primary non-function, acute rejection, ischaemic-type biliary lesions and morbidity in ABO-incompatible liver transplantation.

Another promising area could be the use of macrophage therapy to treat liver disease. Macrophages reduce scar tissue and stimulate the HPCs to expand and differentiate into mature hepatocytes. Thomas et al. showed that bone marrow-derived macrophages (BMM) administered to mice with advanced liver fibrosis resulted in a degradation of fibrillar collagen and reduced fibrogenesis. There was also upregulation of the liver progenitor cell mitogen tumour necrosis factor-like weak inducer of apoptosis that was associated with an expansion of the progenitor cell compartment [97]. There are ongoing clinical trials to assess the role macrophage therapy could play in liver cirrhosis [93]. Significant advances have been made to translate the use of stem cells to promote liver regeneration and mature hepatic differentiation into clinical use. Currently, most trials are in early phase I/II and results demonstrating the efficacy of these techniques are yet to be published. In the near future, the full potential of stem cells for liver regeneration in patients with liver disease may be better established.

## Conclusion

HT offers an alternative therapy to OLT with the aim of treating liver-based metabolic diseases or ALF. Advances in liver cell therapy are being researched to overcome the obstacles associated with HT, particularly the shortage of healthy donor hepatocytes. Although HLCs are promising, no alternative cell source can yet replace the functionality and efficacy of primary human hepatocytes. MSCs likely hold the greatest attribute as immunomodulators, and co-culturing with mature donor hepatocytes. More clinical trials assessing the safety and efficacy of HLCs is pivotal before they can be considered as a reliable cell source. In the future, this may allow for liver-based diseases to be effectively treated without the need for OLT.

## Abbreviations

ALF: Acute liver failure
BMM: Bone marrow-derived macrophages
BMP4: Bone morphogenetic protein 4
CN-1: Crigler-Najjar syndrome type 1
CYP: Cytochrome p450
DEX: Dexamethasone
EGF: Epidermal growth factor
fVII: Factor VII
FGF: Fibroblast growth factor
FHF: Fulminant hepatic failure
hAECs: Hepatic differentiation of amniotic epithelial cells
HPCs: Hepatic progenitor cells
HNF1A: Hepatocyte nuclear factor 1 homeobox alpha
HT: Hepatocyte transplantation
HGF: Hepatocyte-growth factor
HLCs: Hepatocyte-like cells
iPSCs: Induced pluripotent stem cells
PE: Plasma exchange
EpCAM^+^: Primary human bile duct cells
CLiPs: Proliferative bipotent cells
MSCs: Mesenchymal stem cells
MSC-CM: MSC-conditioned medium
OSM: Oncostatin M
OTC: Ornithine transcarbamylase deficiency
OLT: Orthotopic liver transplantation
UC-MSC: Umbilical cord–mesenchymal stem cell
